# Analysis of transmitted drug resistance and HIV-1 subtypes using dried serum spots of recently HIV-infected individuals in 2013 in Germany

**DOI:** 10.7448/IAS.17.4.19670

**Published:** 2014-11-02

**Authors:** Hauser Andrea, Hofmann Alexandra, Santos-Hoevener Claudia, Zimmermann Ruth, Hamouda Osamah, Bannert Norbert, Kuecherer Claudia

**Affiliations:** 1 HIV and Other Retroviruses, Robert Koch Institute, Berlin, Germany; 2 HIV/AIDS and Other Blood Borne Infections, Robert Koch Institute, Berlin, Germany

## Abstract

**Introduction:**

The Robert Koch Institute (RKI) aimed to assess a molecular surveillance strategy based on filter-dried serum spots (DSS) of all newly diagnosed HIV infections in Germany. In 2013, diagnostic laboratories sent DSS to the RKI representing 55% of the newly diagnosed HIV infections reported to the RKI (protection against infection act). DSS were first tested serologically to identify recently acquired infections (<140 days duration of infection); those classified as “recent infection” were processed for HIV-1 genotyping. The aim of this study was to assess the level of TDR and the current HIV-1 subtypes in the main HIV transmission group categories (TrGrpC) in 2013: men who have sex with men (MSM), women/men with heterosexual contacts (HET) and injecting drug users (IDUs).

**Materials and Methods:**

DSS were tested for recency of infection using the BED capture EIA. Viral RNA from “recent infections” was amplified by HIV-1 group M generic pol-RT-PCR covering all resistance-associated positions in the HIV-1 protease (AS1-99) and reverse transcriptase (AS1-252) if viral loads were ≥6,500 copies/mL. PCR amplicons were sequenced (Sanger) to analyze genotypic resistance and the HIV-1 subtype. Results were merged to data from the HIV report, i.e. the TrGrpC.

**Results:**

In 2013, 1027 DSS were classified as recent HIV infections (506 MSM, 118 HET, 31 IDUs, 6 others, 366 unknown). RNA was extracted from 703 recent cases and 389/503 samples with sufficient viral load were PCR-positive. By June 2014, 276/389 samples were sequenced: TDR was identified in 13% (35/276) of the recent infections including single (PI, NRTI, NNRTI) and dual drug class resistant strains (NRTI/NNRTI; NNRTI/PI). 18% (51/276) of recent HIV-1 infections were caused by non-B subtypes (A1, C, CRF01_AE, CRF02_AG, D, F, G, URFs). TDR was observed at comparable levels in all TrGrpC. Proportions of non-B infections were significantly higher in HET (78%; 14/18) and IDUs (60%; 3/5) compared to MSM (8%; 14/169) (p<0.01).

**Conclusions:**

The proportion of TDR was similar but the proportion of HIV-1 subtype non-B infections was higher as previously described for Germany based on results from the German HIV-1 Seroconverter Cohort [1,2]. This difference could be the result of a broadened inclusion of HET and IDUs due to the sampling method used making this study representative for molecular surveillance of HIV-1 in Germany.
